# HBK-17, a 5-HT_1A_ Receptor Ligand With Anxiolytic-Like Activity, Preferentially Activates ß-Arrestin Signaling

**DOI:** 10.3389/fphar.2018.01146

**Published:** 2018-10-16

**Authors:** Karolina Pytka, Monika Głuch-Lutwin, Elżbieta Żmudzka, Kinga Sałaciak, Agata Siwek, Katarzyna Niemczyk, Maria Walczak, Magdalena Smolik, Adrian Olczyk, Adam Gałuszka, Jarosław Śmieja, Barbara Filipek, Jacek Sapa, Marcin Kołaczkowski, Katarzyna Pańczyk, Anna Waszkielewicz, Henryk Marona

**Affiliations:** ^1^Department of Pharmacodynamics, Faculty of Pharmacy, Jagiellonian University Medical College, Krakow, Poland; ^2^Department of Pharmacobiology, Faculty of Pharmacy, Jagiellonian University Medical College, Krakow, Poland; ^3^Chair and Department of Toxicology, Faculty of Pharmacy, Jagiellonian University Medical College, Krakow, Poland; ^4^Control and Robotics Group, Faculty of Automatic Control, Electronics and Computer Science, Institute of Automatic Control, Silesian University of Technology, Gliwice, Poland; ^5^Systems Engineering Group, Faculty of Automatic Control, Electronics and Informatics, Institute of Automatic Control, Silesian University of Technology, Gliwice, Poland; ^6^Department of Medicinal Chemistry, Faculty of Pharmacy, Jagiellonian University Medical College, Krakow, Poland; ^7^Department of Bioorganic Chemistry, Chair of Organic Chemistry, Faculty of Pharmacy, Jagiellonian University Medical College, Krakow, Poland

**Keywords:** 5-HT_1A_ receptor, anxiolytic-like, mouse models, pharmacokinetics, ß-arrestin signaling

## Abstract

Numerous studies have proven that both stimulation and blockade of 5-HT_1A_ and the blockade of 5-HT_7_ receptors might cause the anxiolytic-like effects. Biased agonists selectively activate specific signaling pathways. Therefore, they might offer novel treatment strategies. In this study, we investigated the anxiolytic-like activity, as well as the possible mechanism of action of 1-[(2,5-dimethylphenoxy)propyl]-4-(2-methoxyphenyl)piperazine hydrochloride (HBK-17). In our previous experiments, HBK-17 showed high affinity for 5-HT_1A_ and 5-HT_7_ receptors and antidepressant-like properties. We performed the four plate test and the elevated plus maze test to determine anxiolytic-like activity. Toward a better understanding of the pharmacological properties of HBK-17 we used various functional assays to determine its intrinsic activity at 5-HT_1A_, 5-HT_2A_, 5-HT_7_, and D_2_ receptors and UHPLC-MS/MS method to evaluate its pharmacokinetic profile. We observed the anxiolytic-like activity of HBK-17 in both behavioral tests and the effect was reversed by the pretreatment with WAY-100635, which proves that 5-HT_1A_ receptor activation was essential for the anxiolytic-like effect. Moreover, the compound moderately antagonized D_2_, weakly 5-HT_7_ and very weakly 5-HT_2A_ receptors. We demonstrated that HBK-17 preferentially activated ß-arrestin signaling after binding to the 5-HT_1A_ receptor. HBK-17 was rapidly absorbed after intraperitoneal administration and had a half-life of about 150 min. HBK-17 slightly penetrated the peripheral compartment and showed bioavailability of approximately 45%. The unique pharmacological profile of HBK-17 encourages further experiments to understand its mechanism of action fully.

## Introduction

Brain serotonin participates in numerous physiological and pathological processes. It regulates mood, sleep, or cognitive function and modulates fear and anxiety processes. Serotonin exerts its effects interacting with at least 14 serotonin receptor subtypes. Among all of them, the 5-HT_1A_ receptor plays the most important role in the etiology of anxiety (reviewed by Akimova et al., 2009). The 5-HT_1A_ receptors are expressed both presynaptically and postsynaptically. The activation of presynaptic 5-HT_1A_ autoreceptors, present on serotonergic neurons in the raphe nuclei, reduces serotonergic neurons firing and decreases serotonin levels. On the other hand, the stimulation of postsynaptic 5-HT_1A_ receptors, located mainly on glutamatergic and GABAergic pyramidal neurons, modulates serotonergic sensitivity and participates in emotional and cognitive processes (for review see Garcia-Garcia et al., 2014).

Garcia-Garcia et al. (2014) in their review suggested that inhibiting serotonergic neuron firing and decreasing serotonin release, as well as reducing serotonergic signaling at postsynaptic target receptors might result in anxiolytic-like effects. Studies confirmed this theory, as stimulating 5-HT_1A_ receptors in the dorsal raphe nucleus caused an anxiolytic-like effect (Cervo et al., 2000), whereas activating the heteroreceptors in the medial septum and dorsal hippocampus was anxiogenic (File et al., 1996; De Almeida et al., 1998). Collinson and Dawson (1997) presented the anxiolytic-like effect of 5-HT_1A_ receptor agonist, 8-OH-DPAT, which was reversed by 5-HT_1A_ receptor antagonist (WAY-100635). Moreover, 5-HT_1A_ knockout mice, lacking 5-HT_1A_ auto- and heteroreceptors, displayed a heightened anxiety-like phenotype in several tests in rodents (Heisler et al., 1998; Parks et al., 1998; Ramboz et al., 1998) and resistance to benzodiazepines (Sibille et al., 2000). Overall, the above findings demonstrate the involvement of 5-HT_1A_ receptors in anxiogenic processes.

The blockade of 5-HT_7_ receptors might also be beneficial in anxiety. Yau et al. (2001) showed an increase in 5-HT_7_ receptor mRNA expression after acute, but not chronic, stress in the rat hippocampus, which suggested the role of the 5-HT_7_ receptor in stress regulation. Wesołowska et al. (2006b) demonstrated the anxiolytic-like activity of a selective 5-HT_7_ receptor antagonist, SB-269970, in rodent models of anxiety. Similarly, intrahippocampal administration of the compound induced an antianxiety-like effect in the Vogel conflict test in rats (Wesołowska et al., 2006a). Thus, some studies suggest that 5-HT7 receptor antagonists might decrease anxiety symptoms.

Functional selectivity (also called biased signaling) is an ability of a ligand to direct a G protein-coupled receptor toward a conformation that activates specific signal transduction pathway. Upon stimulation, the 5-HT_1A_ receptor can activate many signaling pathways, including adenylate cyclase inhibition, extracellular signal-regulated protein kinase (ERK) phosphorylation, G protein-coupled inwardly rectifying potassium channel activation, voltage-gated calcium channel inhibition, or the recruitment of ß-arrestin (Chilmonczyk et al., 2015; Stroth et al., 2015). Newman-Tancredi et al. (2009) presented compounds highly selective for a 5-HT_1A_ receptor with biased agonist profiles, i.e., F15599 or F13714. F15599 showed a marked potency for ERK1/2 phosphorylation, which might underlie its potent antidepressant-like activity in rats. On the other hand, F13714, which presented lower potency for ERK1/2 phosphorylation (Newman-Tancredi, 2011), showed an exceptionally potent antidyskinetic activity in rats (Iderberg et al., 2015). This demonstrates that biased agonists at the 5-HT_1A_ receptor might present different pharmacological activity. Although it is not yet clear to what extent functional selectivity can be exploited for therapeutic advantage, some clinically used drugs, e.g., carvedilol (ß-blocker) show this effect (Patel et al., 2010; Kenakin, 2011). Moreover, scientists suggest that functional selectivity can explain the unique features of the antipsychotic drug – aripiprazole (Mailman and Murthy, 2010). Since biased agonists preferentially activate one pathway over others, they might offer novel treatment strategies, i.e., show pharmacological activity without inducing unwanted effects. Thus, compounds with functional selectivity might show interesting pharmacological properties *in vivo* and therefore are worth investigating.

**FIGURE 1:**
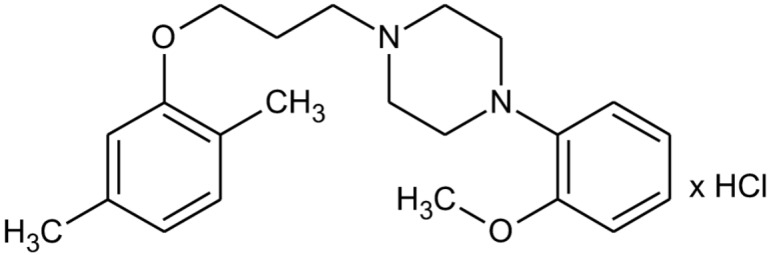
Chemical structure of HBK-17.

We previously demonstrated antidepressant-like activity of 1-[(2,5-dimethylphenoxy)propyl]-4-(2-methoxyphenyl)piperazine hydrochloride (HBK-17, **Figure 1**) in rodents (Waszkielewicz et al., 2015; Kubacka et al., 2016; Pytka et al., 2016a). The compound moderately antagonized 5-HT_1A_ receptors expressed in CHO-K1 cells in Ca^2+^ mobilization assay and showed the affinity for 5-HT_7_ receptors (Waszkielewicz et al., 2015; Kubacka et al., 2016; Pytka et al., 2016a). Since 5-HT_1A_ and 5-HT_7_ receptors play a role in anxiety, we hypothesized that HBK-17 might influence anxiety-like behaviors in rodents. Therefore, our study aimed to investigate anxiolytic-like properties in mice, as well as the possible mechanism of action of HBK-17 using *in vitro* and *in vivo* experiments. As knowledge of pharmacokinetics and brain distribution of novel central-acting compounds is essential for the proper analysis of their *in vivo* effects, we also evaluated the compound’s pharmacokinetic profile.

## Materials and Methods

### Animals

We used adult male Albino-Swiss mice (CD-1) weighing 18–21 g in all experiments. The animals were kept in groups of 15 mice in cages at room temperature of 22 ± 2°C under light/dark (12:12) cycle and they had free access to food (standard laboratory pellets) and water before experiments. Humidity and ambient temperature of the room were kept constant throughout all tests, which were conducted between 9 a.m. and 4 p.m. The animals were used only once in each test. All injections were given in a volume of 10 ml/kg. Behavioral experiments were carried out by a trained observer blind to the treatments. All experimental procedures were approved by the I Local Ethics Committee for Experiments on Animals of the Jagiellonian University in Krakow (approval number 51/2015), Poland and performed under the guidelines provided by the European Union Directive of 22 September 2010 (2010/63/EU) and Polish legislation concerning animal experimentation.

### Drugs

The tested compound 1-[(2,5-dimethylphenoxy)propyl]-4-(2-methoxyphenyl)piperazine hydrochloride (HBK-17) was synthesized in the Department of Bioorganic Chemistry, Chair of Organic Chemistry, Jagiellonian University Medical College (**Figure 1**; Waszkielewicz et al., 2015). HBK-17 was dissolved in saline and administered intraperitoneally (*i.p.*) 30 min before behavioral experiments. p-Chlorophenylalanine (pCPA, Sigma, Germany) and α-methyl-p-tyrosine (AMPT, Sigma, Germany) were suspended in 1% Tween and administered *i.p*. WAY-100635 (Sigma, Germany) was injected subcutaneously (s.c.) 15 min before the studied compound.

### Radioligand Binding Assays

Binding experiments were performed using membranes from CHO-K1 cells stably transfected with the human D_2_ receptor and rat cerebral cortex (α_2_-adrenergic and GABA_A_ receptors). [^3^H]-Methylspiperon (final concentration 0.4 nM; D_2_ receptor), [^3^H]-clonidine (final concentration 0.2 nM; α_2_-adrenergic receptor) and [^3^H]-muscimol (final concentration 1 nM; GABA_A_ receptor) were used. The final incubation mixture (total volume 250 μl) consisted of a 50 μl solution of the test compound, 50 μl of radioligand and 150 μl of diluted membranes or the tissue suspension. The incubation was terminated by rapid filtration over glass fiber filters GF/B (PerkinElmer, United States) using 96-well FilterMate Harvester (PerkinElmer, United States). Five rapid washes were performed with ice-cold 50 mM Tris–HCl buffer (pH 7.4 or 7.6). The radioactivity was measured in MicroBeta TriLux 1450 – liquid scintillation counter (PerkinElmer, United States). Radioligand binding data were analyzed using iterative curve fitting routines GraphPad Prism 5.0 (GraphPad Software). Ki values were calculated from the Cheng and Prusoff (1973) equation. The concentrations of analyzed compounds ranged from 10^−10^ to 10^−5^ M. For measuring unspecific binding, clonidine – 10 μM (in case of [^3^H]-clonidine) and haloperidol – 1 μM (in case of [^3^H]-methylspiperon) and diazepam – 100 μM (in case of [^3^H]-muscimol) were applied. At least two independent experiments were performed for each assay.

### Functional Assays

Serial dilutions were prepared in 96-well microplate in appropriate dilution buffer (HBSS or medium) with 0.1% BSA added, and 8–10 concentrations were tested in duplicates. At least two independent experiments were performed for each type of method. Assays were done according to manufacturer’s protocols.

The level of cAMP (TRF0263, PerkinElmer assay kit) was monitored using CHO-K1 cells with expression of the human serotonin 5-HT_1A_ receptor. The cells were resuspended in stimulation buffer at 2 × 10^5^ cells/ml. The same volume (10 μl) of cells was added to tested compounds with 10 μM forskolin. The antagonist response was performed using as a reference agonist, serotonin, in EC_80_ (120 nM). Samples were loaded onto a white opaque half area 96-well microplate, incubated for 40 min at room temperature. The 10 μl of reagents were added, mixed, and incubated for 1 h. The homogeneous TR-FRET signal was read on an EnVision Microplate Reader (PerkinElmer, United States).

The CHO-5HT1A receptor cells were tested for phosphorylated-ERK (p-ERK) using the SureFire ERK-Phosphorylation AlphaLISA Assay Kit according to the manufacturer’s instruction (ALSU-PERK-A10K, PerkinElmer). At the experiment, cells were plated at 50,000 cells/well of 96-well plate and grown 7 h in an incubator (5% CO_2_, 37°C). The serial dilutions of compounds were added and incubated for 15 min at 37°C. The antagonist response was performed using serotonin as a reference agonist at the concentration equal to EC_80_ (45 nM). The “lysis buffer” (70 μl) was added and the plate gently agitated on a plate shaker (10 min). The 10 μl of samples were transferred to assay plates (384-OptiPlate, PerkinElmer) in duplicates and 10 μl of the reaction mix was added. The plates were incubated for 2 h at 22°C and measured with an EnVision a multifunction plate reader (PerkinElmer Life Science).

The HTR1A-bla U2OS receptor cells were tested using the Tango LiveBLAzer β-arrestin assay kit according to the manufacturer’s instruction (K1095, Life Technologies). At the experiment, cells were plated at 10,000 cells/well of 384-well black, clear bottom plate and grown 12 h in an incubator (5% CO_2_, 37°C). The serial dilutions of compounds were added and incubated for 5 h (5% CO_2_, 37°). The antagonist response was performed using serotonin as a reference agonist at the concentration equal to EC_80_ (300 nM). After this time, 8 μl of the reaction mix was added. The plates were incubated for 2 h at 22°C and measured with a FLUOstar OPTIMA a multifunction plate reader (PerkinElmer Life Science).

### Behavioral Experiments

#### Four Plate Test

The four plate test was performed on mice according to the method previously described (Aron et al., 1971; Bourin et al., 2005; Pytka et al., 2016b). Mice were placed individually in the four plate apparatus connected to the power source. After a 15 s of habituation period, each mouse crossing from one plate to another (two limbs on one plate, two on another) was punished by an electric shock (0.8 mA, 0.5 s). If the mouse continued running, it received no new shock for the following 3 s. The number of punished crossings was calculated during the 60 s of the test. HBK-17 was dissolved in saline and administered *i.p.* 30 min before the test.

#### Elevated Plus Maze

The elevated plus maze was performed according to the method previously described (Lister, 1987; Pytka et al., 2017). The elevated plus maze for mice consisted of two opposing open (30 cm × 5 cm), and two enclosed arms (30 cm × 5 cm × 25 cm) connected by a central platform forming the shape of a plus sign. The open and closed arms were connected with a central field (5 cm × 5 cm). Each mouse was individually placed at the central field of the apparatus with the head turned toward one of the closed arms. Animal behavior was observed for 5 min. The device was disinfected with 70% ethanol after each mouse. The number of entries to open and closed arms and time spent in the open and closed arms were measured. The experiments were recorded and scored using aLab.io software by a trained observer blind to the treatments. HBK-17 was dissolved in saline and administered *i.p.* 30 min before the test.

#### Spontaneous Locomotor Activity

Spontaneous locomotor activity was performed as previously described (Pytka et al., 2015a). Photoresistor actometers (Ugo Basile, Italy) connected to a counter for the recording of light-beam interruptions were used to investigate the effect of the compound on locomotor activity in mice. Locomotor activity was counted as the number of the light-beam crossing. It was recorded individually for each animal. After administration of the tested compound, each mouse was placed separately in a cage for 30 min habituation period. After that time, the parameter was measured for 1 min and 5 min (i.e., time equal to the observation period in four plate test and elevated plus maze test, respectively). The cages were sanitized with 70% ethanol after each mouse. HBK-17 was dissolved in saline and administered *i.p.* 30 min before the test.

#### Hot Plate Test

The hot plate test was performed as described by Sałat et al. (2015). The hot plate apparatus (Hot/Cold Plate, Bioseb, France) consisted of an electrically heated surface and was equipped with a temperature controller that keeps the temperature constant at 55–56°C. One day before the experiment, the animals were tested for their pain sensitivity threshold (baseline latency). We chose only mice that showed baseline latencies ≤30 s. The latency time to pain reaction (licking hind paws or jumping) was measured as the indicative of nociception (cutoff time – 60 s). Animals that did not respond within 60 s were removed from the hot plate apparatus and assigned a score of 60 s. HBK-17 was dissolved in saline and administered *i.p.* 30 min before the test.

#### Serotonin Synthesis Blockade

To assess the involvement of the serotonergic system in the anxiolytic-like effect of HBK-17, we used pCPA (tryptophan hydroxylase inhibitor) as previously described (Pytka et al., 2015b). We injected mice with pCPA at a dose of 200 mg/kg for three consecutive days. 24 h after the last pCPA administration, we injected mice with saline (*i.p.*) or HBK-17 (5 mg/kg, *i.p.*). We performed the four plate test 30 min after the administration.

#### Noradrenaline and Dopamine Synthesis Blockade

To investigate the involvement of the noradrenergic and dopaminergic systems in the anxiolytic-like effect of HBK-17, we pretreated mice *i.p.* with a catecholamine synthesis inhibitor, AMPT (tyrosine hydroxylase inhibitor) at the dose of 100 mg/kg. Control group received vehicle (1% Tween). Four hours after AMPT or 1% Tween injection, mice were pretreated with saline (*i.p.*) or HBK-17 (5 mg/kg, *i.p.*). We performed the four plate test 30 min after the compound administration.

### Pharmacokinetic Experiments

#### Instrumentation and Bioanalysis

The LC/ESI-MS/MS experiments were performed on TSQ Quantum Triple Quadrupole Mass Spectrometer (Thermo Scientific, United States) equipped with an electrospray ionization interface. This instrument was coupled to Dionex 3000 (Dionex, United States) ULPC system. Data acquisition and processing were accomplished using Xcalibur data collection and integration software. The mobile phase consisted of a mixture of acetonitrile with an addition of 0.1% formic acid (Solvent A) and water with an addition of 0.1% formic acid (Solvent B) was set at a flow rate of 0.3 ml/min in gradient elution. Sample preparations were carried out by precipitation procedure using acetonitrile after the addition of the internal standard [IS, 2-(4-methyl-1-piperazinyl)-4-phenylquinazoline]. The dried residue was reconstituted in the mobile phase and injected onto an Acclaim Polar Advantage Column (1.8 μm, 100 mm × 2.1 mm, Dionex).

The method was validated according to validation procedures, parameters and acceptance criteria based on USP XXIII guidelines and FDA criterion 20/15.

#### Pharmacokinetic Study in Mice

All data in pharmacokinetic experiments were processed with the pharmacokinetics software Phoenix WinNonlin (Certara Company, United States). The non-compartmental pharmacokinetic parameters of t_1/2_, MRT, AUC, Cl, and V_d_ were calculated based on moment methods. First order elimination rate constant (λ_z_) was calculated by linear regression of time vs. log concentration according to Eq. 1.

(1) $$\lambda_{z}=-2.303a$$

where a is a slope of a line.

The terminal half-life (t_1/2_) was calculated as:

(2) $$t_{1/2}=\frac{0.693}{\lambda_{z}}$$

The area under curve the mean plasma and tissue concentration vs. time curve extrapolated to infinity (AUC_0→∞_) was estimated using the log-linear trapezoidal rule (Eq. 3), here C_n_ is the concentration of last sampling.

(3) $${\mathrm{AUC}}_{0\to \infty }=\sum_{i=1}^{n}\left(\frac{C_{i}+C_{i+1}}{2}\right)\cdot (t_{i+1}-t_{i})+\frac{C_{n}}{\lambda_{z}}$$

Area under the first-moment curve (AUMC_0→∞_) was estimated by calculation of total area under the first-moment curve and extrapolated area using the Eq. 4, where t_n_ is the time of last sampling.

(4) $$\begin{array}{lll} {\mathrm{AUMC}}_{0\to \infty } & = & \sum_{i=1}^{n}\left(\frac{t_{i}\cdot C_{i}+t_{i+1}\cdot C_{i+1}}{2}\right)\cdot (t_{i+1}-t_{i})\\ & & +\frac{t_{n}\cdot C_{n}}{\lambda_{z}}+\frac{C_{n}}{\lambda_{z}} \end{array}$$

Mean residence time (MRT) was calculated as:

(5) $$\mathrm{MRT}=\frac{{\mathrm{AUMC}}_{0\to \infty }}{{\mathrm{AUC}}_{0\to \infty }}$$

Systemic clearance (Cl) was calculated as:

(6) $$\mathrm{Cl}=\frac{D_{\mathrm{iv}}}{{\mathrm{AUMC}}_{0\to \infty }}$$

Volume of distribution (V_d_) was calculated as:

(7) $$V_{d}=\frac{D_{\mathrm{iv}}}{C_{0}}$$

were D_iv_ is an intravenous dose and C_0_ is an initial concentration.

The bioavailability (F) of the studied compound after an intraperitoneal administration compared to the intravenous route was calculated as:

(8) $$F=\frac{{\mathrm{AUC}}_{\mathrm{i.p.}}\cdot D_{\mathrm{i.v.}}}{{\mathrm{AUC}}_{\mathrm{i.v.}}\cdot D_{\mathrm{i.p.}}}\cdot 100\%$$

### Data Analysis

Results are presented as means ± SEM. They were estimated using one or two-way analysis of variance (ANOVA), followed by Newman-Keuls or Bonferroni *post hoc*, respectively. Differences between groups were considered as significant if *p* < 0.05.

## Results

### HBK-17 Moderately Antagonized D_2_ and Weakly 5-HT_7_ Receptors

The radioligand binding experiments revealed that compared with the reference compound, haloperidol, HBK-17 moderately bound to D_2_ receptors. HBK-17 did not show the affinity for α_2_ adrenoceptors or GABA_A_ receptors, while the reference drugs clonidine and diazepam strongly bound to these receptors, respectively (**Table 1**).

**Table 1:** The affinity of HBK-17 for adrenergic α_2_, dopaminergic D_2_, and GABAergic GABA_A_ receptors.

Compound	α_2_ receptors K_i_ ± SEM [nM]	D_2_ receptors K_i_ ± SEM [nM]	GABA_A_ receptors K_i_ ± SEM [nM]
HBK-17	17800 ± 1.5	51.4 ± 0.7	>10000
Clonidine	2.5 ± 0.1	–	–
Haloperidol	–	1.1 ± 0.1	–
Diazepam	–	–	5.6 ± 0.7

*Inhibition constants (K_i_) were calculated according to the equation of Cheng and Prusoff (1973).*

Since HBK-17 showed a moderate affinity for D_2_, 5-HT_2A_, and 5-HT_7_ receptors, we investigated its intrinsic activity toward these receptors. The results of the functional assays showed that HBK-17 had no agonistic properties, but it blocked dopamine D_2_ receptor. Its value of the equilibrium dissociation constant for a competitive antagonist (K_b_) was 7.4-fold lower than the K_b_ value of the reference antagonist – chlorpromazine (**Table 2**). Similarly, HBK-17 did not show any agonistic properties at serotonin 5-HT_7_ receptor but blocked the receptor with K_b_ value 78-fold lower than the reference antagonist, SB269970 (**Table 2**). As demonstrated in **Table 2** HBK-17 showed no agonistic properties and negligible antagonistic effect at serotonin 5-HT_2A_ receptor. Compared with the reference compound, α-methylserotonin, HBK-17 blocked 5-HT_2A_ receptor 1228.1-fold weaker (**Table 2**).

**Table 2:** Intrinsic activity of HBK-17 toward 5-HT_2A_, 5-HT_7_, and D_2_ receptors.

Receptor	Treatment	Agonist mode^∗^			Antagonist mode^∗∗^				
		E_max_ %	SEM	pEC_50_	E_max_ %	SEM	pIC_50_	K_b_ [nM]	R^2^ K_b_
5-HT_7_	Serotonin	100%	3.5	8.34	1%	7.0	n.c.	n.c.	n.c.
	SB269970	4%	3.5	n.c.	8%	0.0	8.82	0.5	0.993
	HBK-17	18%	1.5	n.c.	11%	0.0	6.91	39	0.919
5-HT_2A_	α-Methylserotonin	100%	0.0	8.70	1%	0.0	n.c.	n.c.	n.c.
	Pimavanserin	1%	0.5	n.c.	0%	0.0	8.04	0.57	0.948
	HBK-17	1%	0.0	n.c.	58%	3.0	4.95	700	0.932
D_2_	Apomorphine	100%	2.0	7.89	1%	0.5	n.c.	n.c.	n.c.
	Chlorpromazine	5%	2.5	n.c.	1%	0.0	8.53	0.89	0.956
	HBK-17	2%	0.0	n.c.	0%	0.0	7.65	6.63	0.881

*E_max_, the maximum possible effect; pEC_50_, the negative logarithm of concentration of compound where 50% of its maximal effect was observed; pIC_50_, the negative logarithm of concentration of a compound where 50% of its maximal inhibitory effect was observed; K_b_, the equilibrium dissociation constant for a competitive antagonist determined using of the Cheng-Prusoff equation; n.c., not calculable. ^∗^Results were normalized as percentage of maximal agonist response (5-HT_2A_, α-methylserotonin 10^−6^ M; 5-HT_7_, serotonin 10^−6^ M; D_2_ receptors, apomorphine 10^−6^ M). ^∗∗^Results were normalized as percentage of maximal response in the absence of antagonist.*

### HBK-17 Preferentially Activated ß-Arrestin Recruitment After Binding to 5-HT_1A_ Receptor

A single receptor can activate numerous signaling pathways and influence cell function oppositely. Biased agonists stabilize the receptor’s conformation preferentially, and consequently activate a selected signaling pathway. In our study we used cell cultures as they provide a valuable complement to *in vivo* experiments, allowing more controlled manipulation of the cellular functions and processes.

Although 5-HT_1A_ receptor couples to the broad panel of second messengers, the primary coupling linkage is to the inhibition of adenylate cyclase (Chilmonczyk et al., 2015). Therefore, first, we evaluated the influence of HBK-17 on cAMP production using CHO-K1 cells with expression of the human serotonin 5-HT_1A_ receptor. Contrary to the reference compound, serotonin, HBK-17 did not inhibit cAMP formation. However, the studied compound showed antagonistic properties in this assay. HBK-17 demonstrated 6.1-fold lower K_b_ value compared with the reference compound, WAY-100135 (**Table 3**).

**Table 3:** Intrinsic activity of HBK-17 at 5-HT_1A_ receptor in various functional assays.

5-HT_1A_ assay	Treatment	Agonist mode^∗^			Antagonist mode^∗∗^				
		E_max_ %	SEM	pEC_50_	E_max_ %	SEM	pIC_50_	K_b_ [nM]	R^2^ K_b_
cAMP	Serotonin	100	2.0	7.52	4	0.5	n.c.	n.c.	n.c.
	WAY-100135	2	0.5	n.c.	8	1.0	7.9	6.4	0.939
	HBK-17	1	1.5	n.c.	4	1.0	6.8	39	0.894
ß-arrestin	Serotonin	100	0.5	7.13	2	1.0	n.c	n.c.	n.c.
	WAY-100135	1	0.5	n.c.	5	0.9	8.1	5.9	0.779
	HBK-17	94	0.5	6.49	29	3.0	n.c.	n.c.	n.c.
pERK1/2	Serotonin	100	5.0	7.95	1	0.5	n.c	n.c.	n.c.
	WAY-100135	2	1	n.c.	0	0.0	7.90	2.7	0.769
	HBK-17	40	1.5	7.85	35	0.9	6.7	29	0.836
Ca^2+^	Serotonin	100^*a*^	1.0^*a*^	6.50^*a*^	1	0.0	n.c.^*a*^	n.c.^*a*^	n.c.^*a*^
	WAY-100135	2	0	n.c.	0	0.0	8.3	0.8	0.997
	HBK-17	1^*a*^	0.0^*a*^	n.c^*a*^	4^*a*^	0.0^*a*^	8^*a*^	6.9^*a*^	0.907^*a*^

*E_max_, the maximum possible effect; pEC_50_, the negative logarithm of concentration of compound where 50% of its maximal effect was observed; pIC_50_, the negative logarithm of concentration of a compound where 50% of its maximal inhibitory effect was observed; K_b_, the equilibrium dissociation constant for a competitive antagonist determined using of the Cheng-Prusoff equation; n.c., not calculable. ^∗^Results were normalized as percentage of maximal agonist response (serotonin 10^−6^ M). ^∗∗^Results were normalized as percentage of maximal response in the absence of antagonist. ^a^Kubacka et al. (2016).*

To prevent over-stimulation G protein-coupled receptors recruit cytosolic proteins – arrestins – which promote signaling termination. Thus, we determined the influence of HBK-17 on β-arrestin recruitment using HTR1A-bla U2OS receptor cells. The functional studies revealed that HBK-17 relative to serotonin moderately activated ß-arrestin recruitment in 5-HT_1A_ receptor (**Table 3**). As presented in **Table 3** the efficacy of HBK-17 (*E*_max_ = 94%) was similar relative to that induced by 10 μmol L^−1^ serotonin (*E*_max_ = 100%), but the potency was 4.4-fold lower. In the antagonist mode, HBK-17 did not show any effect in this assay.

Since stimulation of 5-HT_1A_ receptor may lead to the activation of the ERK/MAPK pathway, we determined the effect of HBK-17 on the level p-ERK1/2 using the CHO-5HT1A receptor cells. The compound showed partial agonistic properties in this assay. In the p-ERK1/2 assay, HBK-17 showed 6.6-fold higher potency than serotonin, but 2.5-fold lower efficacy (**Table 3**). In the antagonist mode HBK-17, compared with the reference antagonist, WAY-100135, showed 10.7-fold lower K_b_ value.

Our previous studies demonstrated that in the Ca^2+^ mobilization assay the reference compound, WAY-100135, showed strong antagonistic properties (**Table 3**; Kubacka et al., 2016).

### HBK-17 Increased the Number of Punished Crossings in the Four Plate Test and Did Not Affect the Pain Response in the Hot Plate Test in Mice

**FIGURE 2:**
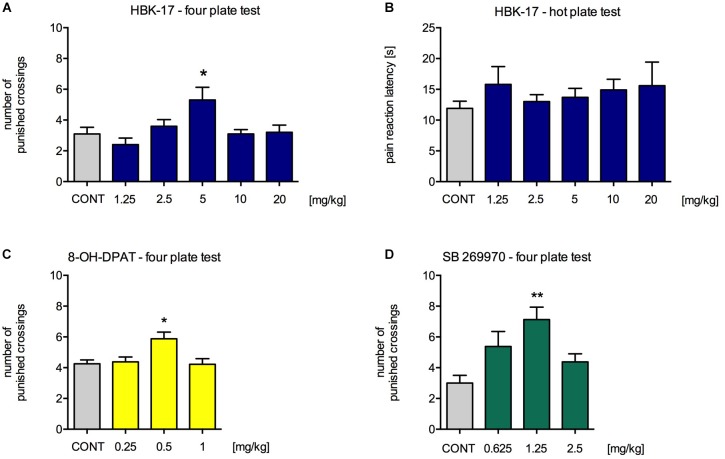
The effect of HBK-17 **(A)**, 8-OH-DPAT **(C)**, and SB 269970 **(D)** on the behavior of mice in the four plate test and the influence of HBK-17 on the pain threshold **(B)**. HBK-17 and SB 269970 were administered intraperitoneally 30 min before the experiment, whereas 8-OH-DPAT was injected 15 min before the four plate test. CONT – control group; Statistical analysis: one-way ANOVA (Newman-Keuls *post hoc*) ^∗^*p* < 0.05, ^∗∗^*p* < 0.01 vs. control group; *n* = 8–10 mice per group.

In order to evaluate the anxiolytic-like properties of the compound, we performed four plate test in mice. The tested compound HBK-17 at a dose 5 mg/kg (but not 1.25, 2.5, 10, or 20 mg/kg) significantly increased the number of punished crossings by 70.9% compared with non-treated control [*F*(5,54) = 3.791, *p* < 0.01] (**Figure 2A**). A reference 5-HT_1A_ receptor agonist, 8-OD-DPAT, at a dose 0.5 mg/kg (but not 0.25, 1 or 2 mg/kg) significantly increased the number of punished crossings by 38.2% [*F*(4,37) = 3.791, *p* < 0.05] (**Figure 2C**). Another reference compound SB269970, a 5-HT_7_ receptor antagonist, at a dose 2.5 mg/kg (but not 1.25 or 5 mg/kg) increased the number of punished crossings by 137.5% compared with non-treated control [*F*(3,29) = 5.903, *p* < 0.01] (**Figure 2D**). Compounds with analgesic properties might show false positive results in the four plate test. Therefore, we evaluated the HBK-17 influence on pain responses in the hot plate test in mice. HBK-17 at doses 1.25–20 mg/kg did not increase the latency time to the first hind paw or/and jumping response [*F*(5,54) = 0.466, ns] (**Figure 2B**).

### HBK-17 Increased the Time in the Open Arms and Open Arm Entries in the Elevated Plus Maze Test in Mice

**FIGURE 3:**
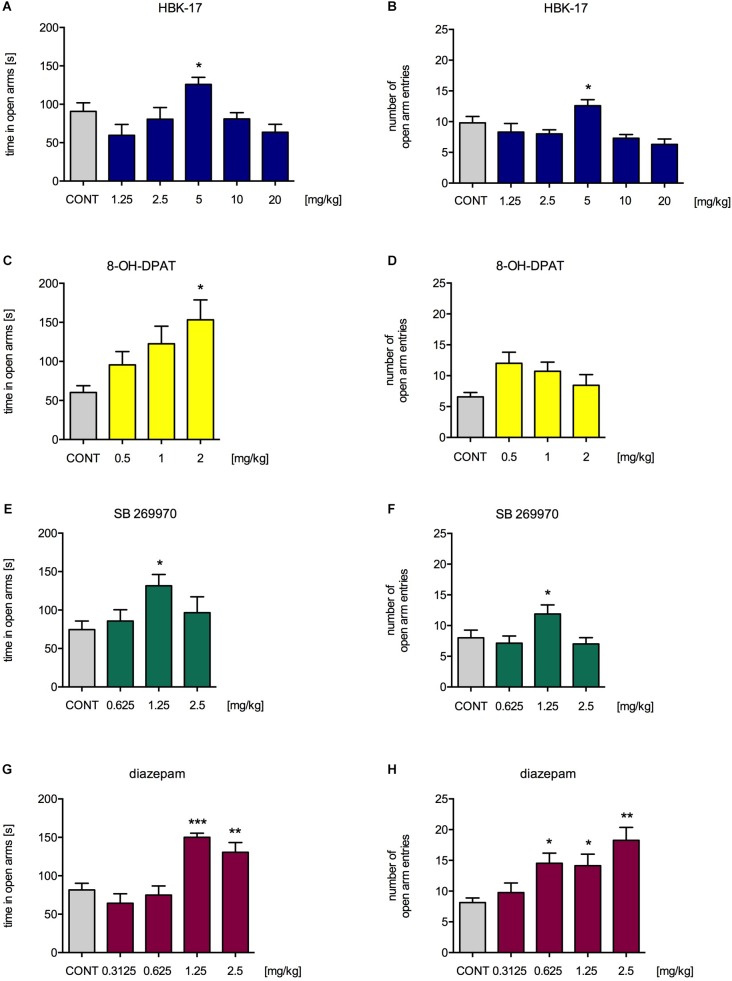
The effect of HBK-17 **(A,B)**, 8-OH-DPAT **(C,D)**, SB 269970 **(E,F)**, or diazepam **(G,H)** on the behavior of mice in the elevated plus maze test. HBK-17, SB 269970, and diazepam were administered intraperitoneally 30 min before the test, whereas 8-OH-DPAT was injected 15 min before the elevated plus maze test. CONT. CONT – control group; Statistical analysis: one-way ANOVA (Newman-Keuls *post hoc*) ^∗^*p* < 0.05, ^∗∗^*p* < 0.01, ^∗∗∗^*p* < 0.001 vs. control group; *n* = 8–10 mice per group.

To confirm our findings, we performed another experiment assessing anxiolytic-like properties of the compound, i.e., elevated plus maze test. HBK-17 at a dose 5 mg/kg (but not 1.25, 2.5, 10, or 20 mg/kg) compared with non-treated control significantly increased the time spent in the open arms by 38.9% [*F*(5,54) = 4.143, *p* < 0.01] and the number of open arm entries by 28.6% [*F*(5,54) = 5.267, *p* < 0.01] (**Figures 3A,B**). 8-OH-DPAT at a dose 2 mg/kg (but not 0.25, 0.5 or 1 mg/kg) compared with non-treated control significantly increased the time spent in the open arms by 154.3% [*F*(3,29) = 3.710, *p* < 0.05] but had no effect on the number of open arm entries [*F*(3,29) = 2.224, ns] (**Figures 3C,D**). SB269970 at a dose 2.5 mg/kg (but not 1.25 or 5 mg/kg) compared with non-treated control significantly increased the time spent in the open arms by 76.4% [*F*(3,28) = 3.026, *p* < 0.05] and the number of open arm entries by 48.6% [*F*(3,30) = 3.396, *p* < 0.05] (**Figures 3E,F**). Diazepam at the doses 1.25 and 2.5 mg/kg (but not 0.3125 or 0.625 mg/kg) compared with non-treated control significantly increased the time spent in the open arms by 84.6 and 59.9% [*F*(4,35) = 12.870, *p* < 0.0001], respectively. The reference compound significantly increased the number of open arm entries at the doses 0.625, 1.25, and 2.5 mg/kg (but not 0.3125 mg/kg) by 78.5, 73.9, and 124.6% [*F*(4,35) = 5.908, *p* < 0.01], respectively (**Figures 3G,H**).

### No Effect of HBK-17 After Pretreatment With pCPA or WAY-100635, But Not AMPT, in the Four Plate Test in Mice

**FIGURE 4:**
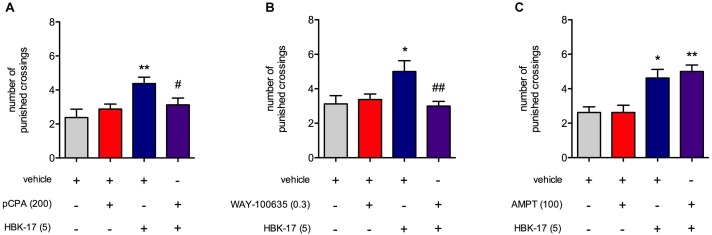
The effect of p-chlorophenylalanine [pCPA, **(A)**], WAY-100635 **(B)**, or α-methylphenylalanine [AMPT, **(C)**] on anxiolytic-like activity of HBK-17 in the four plate test in mice. pCPA was injected intraperitoneally (*i.p.*) once daily for three consecutive days. Twenty-four hour after the last injection and 30 min before the test mice received (*i.p.*) either HBK-17 or 0.9% NaCl. WAY-100635 was administered subcutaneously 15 min before HBK-17. AMPT was injected (*i.p.*) 4 h before HBK-17. Vehicle-treated groups received 1% Tween (*i.p.*). Statistical analysis: two-way ANOVA (Bonferroni *post hoc*); ^∗^*p* < 0.05, ^∗∗^*p* < 0.01 vs. respective control group receiving vehicle or 1% Tween; ^#^*p* < 0.05, ^##^*p* < 0.01 vs. respective group receiving HBK-17 at the dose 5 mg/kg; *n* = 8 mice per group.

To find the possible mechanism of anxiolytic-like activity of HBK-17, we again performed the four plate test, but this time using tryptophan hydroxylase inhibitor (pCPA), tyrosine hydroxylase inhibitor (AMPT), and 5-HT_1A_ receptor antagonist (WAY-100635), which would show the involvement of the serotonergic system, noradrenergic system, and the 5-HT_1A_ receptor, respectively. HBK-17 (5 mg/kg) significantly increased number of punished crossings of mice by 84.2% compared with non-treated control group. Three days treatment with pCPA (200 mg/kg) did not influence the measured parameter but abolished the activity of HBK-17 (**Figure 4A**). The two-way ANOVA demonstrated no effect of pCPA [*F*(1,28) = 0.887; ns], significant effect of HBK-17 [*F*(1,28) = 7.986; *p* < 0.01], and significant interaction [*F*(1,28) = 4.831; *p* < 0.05]. Similarly, the administration of HBK-17 (5 mg/kg) increased the number of punished crossings by 60.0% compared with non-treated control group. Pretreatment with WAY-100635 (0.3 mg/kg) did not influence the number of punished crossings, but it antagonized the effect of HBK-17 (**Figure 4B**). The two-way ANOVA demonstrated no effect of WAY-100635 [*F*(1,28) = 3.092; ns], significant effect of HBK-17 [*F*(1,28) = 4.209; *p* < 0.05], and significant interaction [*F*(1,28) = 8.589; *p* < 0.01]. The injection of HBK-17 (5 mg/kg) caused a significant increase by 76% in the number punished crossings, while AMPT (100 mg/kg) had no effect on this parameter. Pretreatment with AMPT did not influence the effect of HBK-17 (**Figure 4C**) in this test. The two-way ANOVA showed no effect of AMPT [*F*(1,28) = 0.209; ns], significant effect of HBK-17 [*F*(1,28) = 28.490; *p* < 0.05], and no significant interaction [*F*(1,28) = 0.209; ns].

### Neither HBK-17 Alone nor Co-Administered With pCPA, AMPT or WAY-100635 Affected Locomotor Activity in Mice

To investigate whether the observed increased number of punished crossings was not due to psychostimulant properties, we evaluated the effect of HBK-17 on locomotor activity of mice in the 1-min session. The compound did not influence the locomotor activity of mice at the doses 1.25–20 mg/kg during the 1-min session [*F*(5,42) = 2.616; ns] (**Table 4**). To exclude false positive results in the elevated plus maze test, we also evaluated the effect of HBK-17 on locomotor activity of mice in 5-min session. The compound did not influence the locomotor activity of mice at the doses 1.25–20 mg/kg during the 5-min session [*F*(5,42) = 2.485; ns] (**Table 4**). Neither HBK-17 alone nor in combinations with pCPA or AMPT influenced the locomotor activity of mice (**Table 4**). The two-way ANOVA demonstrated no effect of pCPA [*F*(1,28) = 0.405; ns], no effect of HBK-17 [*F*(1,28) = 0.192; ns], and no significant interaction [*F*(1,28) = 1.001; ns]. In case of AMPT the two-way ANOVA showed no effect of AMPT [*F*(1,28) = 0; ns], no effect of HBK-17 [*F*(1,28) = 0.026; ns], and no significant interaction [*F*(1,28) = 1.224; ns]. The administration of HBK-17, WAY-100635 or the combination of both, did not affect the locomotor activity of mice (**Table 4**). The two-way ANOVA demonstrated no effect of WAY-100635 [*F*(1,28) = 1.452; ns], no effect of HBK-17 [*F*(1,28) = 0.088; ns], and no significant interaction [*F*(1,28) = 1.316; ns].

**Table 4:** The influence of HBK-17 and its combinations with pCPA, AMPT, or WAY-100635 on locomotor activity of mice.

Treatment	Dose [mg/kg]	Number of crossings ± SEM		
Vehicle	–	36.0	±	8.3
	1.25	32.6	±	4.7
HBK-17	2.5	35.6	±	6.6
(1-min session)	5	44.8	±	8.4
	10	59.3	±	6.4
	20	31.3	±	3.2
Vehicle	–	323.4	±	53.7
	1.25	295.6	±	20.4
HBK-17	2.5	326.1	±	19.5
(5-min session)	5	317.0	±	24.5
	10	289.9	±	19.5
	20	204.8	±	19.1
Vehicle	–	41.8	±	9.9
HBK-17	5	30.9	±	7.6
pCPA	200	29.4	±	6.8
HBK-17 + pCPA	5 + 200	33.6	±	5.0
Vehicle	–	33.1	±	7.1
HBK-17	5	23.3	±	3.9
AMPT	100	24.5	±	7.3
HBK-17 + AMPT	5 + 100	31.9	±	8.5
Vehicle	–	48.3	±	5.9
HBK-17	5	41.1	±	9.9
WAY-100635	0.3	27.9	±	7.8
HBK-17 + WAY-100635	5 + 0.3	40.4	±	9.9

*HBK-17 was administered intraperitoneally (i.p.) 30 min before the test. pCPA was injected intraperitoneally (i.p.) once daily for three consecutive days. Twenty-four hour after the last injection and 30 min before the test mice received (i.p.) either HBK-17 or 0.9% NaCl. WAY-100635 was administered subcutaneously 15 min before HBK-17. AMPT was injected (i.p.) 4 h before HBK-17. Vehicle-treated groups received 0.9% NaCl (i.p.). Statistical analysis: one-way ANOVA (Newman-Keuls post hoc; in case of HBK-17 administered alone) or two-way ANOVA (Bonferroni post hoc; in case of combined treatments); n = 8 mice per group.*

### Pharmacokinetic Study and Bioavailability

We used a well-validated UPLC-MS/MS method to determine pharmacokinetic parameters after *i.v.* and *i.p.* administration of HBK-17 at a dose of 5 mg/kg in mice. The parameters for the compound are presented in **Table 5**. HBK-17 was eliminated rather fast (*i.v.*: *t*_1/2_ = 105 min; *i.p.*: *t*_1/2_ = 150 min). The absorption of the compound after *i.p.* administration was also fast (*t*_max_ = 5 min) with the maximum plasma concentration at 806.7 ng/mL. The volume of distribution was 9.7 L/kg, suggesting the distribution of the compound throughout the total body water. The absolute bioavailability after *i.p.* administration, which was estimated based on the AUC_0→∞_ calculated from zero time to infinity, was significant (*F* = 44.8%).

**Table 5:** Pharmacokinetic parameters for HBK-17 after *i.v.* and *i.p* administration at a dose of 5 mg/kg to mice.

	Parameters		
	*i.v.*		*i.p.*
C_0_ [ng/mL]	1482		–
AUC_0→∞_ [ng . min/mL]	55558		24877
MRT [min]	107.9		101.4
t_0.5_ [min]	105.4		149.9
C_max_ [ng/mL]	–		806.7
t_max_ [min]	–		5
V_d_ [L/kg]	13683.6		–
Cl [L/min/kg]	90		–
F [%]		44.8	

*C_0_, the initial concentration; C_max_, the maximum plasma concentration; t_max_, time to reach the maximum plasma concentration; t_1/2_, terminal half-life; MRT, mean residence time; AUC_0→∞_, area under the concentration-time curve from zero up to infinitive time; Cl, systemic clearance; V_d_, volume of distribution; F, bioavailability.*

HBK-17 distributed widely and rapidly in the brain within the time course examined. Thus, we can conclude that the compound penetrated the blood-brain barrier. We detected the highest concentrations of HBK-17 5 min after *i.p.* administration. The compound’s concentration in the brain decreased significantly within 8 h, which suggests that HBK-17 not tend to accumulate. The AUC ratio between brain and plasma was 1.13 and 0.96 after *i.v.* or *i.p.* administration revealed a satisfactory binding affinity to mice brain. The other parameters were as follows: AUC_0→∞_ = 23860 ng . min/g, MRT = 147.1 min, *C*_max_ = 523.8 ng/g, *t*_max_ = 5 min.

## Discussion

We found that HBK-17 possessed anxiolytic-like activity in mice, which depended on the 5-HT_1A_ receptor activation. After binding to the 5-HT_1A_ receptor, the compound preferentially activated β-arrestin recruitment over Ca^2+^ mobilization, phosphorylation of ERK1/2 or cAMP formation inhibition. We also demonstrated that following a single *i.v.* or *i.p.* administration, HBK-17 showed relatively high bioavailability, rapid absorption to the blood but rather low distribution to the brain.

Serotonin 5-HT_1A_ and 5-HT_7_ receptors play a role in anxiety (Żmudzka et al., 2018). Studies proved that both stimulation and blockade of 5-HT_1A_ and the blockade of 5-HT_7_ receptors might cause anxiolytic-like effects (Garcia-Garcia et al., 2014; Nikiforuk, 2015). Since HBK-17 showed affinity for both 5-HT_1A_ and 5-HT_7_ receptors, it might also influence anxiety-like behaviors. Thus, as the first step of our studies, we investigated the compound’s anxiolytic-like properties using a preliminary assay based on the unconditioned fear model of anxiety, i.e., the four plate test in mice. Our results show that the compound possessed significant anxiolytic-like activity at one dose tested (5 mg/kg). Since HBK-17 did not influence pain responses and locomotor activity of mice, we can conclude that the obtained results were specific to anxiety.

Next, we decided to confirm our findings in another experiment, i.e., the elevated plus maze, which uses a natural aversion of rodents for open and elevated areas. Compounds with anxiolytic-like properties increase the time spent and the number of entries to the open arms of the elevated plus maze. Similarly, the administration of HBK-17 at only one dose (5 mg/kg) increased the time spent and the number of entries in the open arms of the elevated plus maze. The compound did not affect the locomotor activity of mice; thus, the observed effect could not be attributed to psychostimulant properties. In our previous experiments, HBK-17 showed strong antiarrhythmic properties in adrenaline-induced arrhythmia in rats (Pytka et al., 2016a). Anxiety disorder patients often report autonomic manifestations of anxiety, such as increased heart rate or blood pressure (Gałuszka, 2013). These factors also determine anxiety levels in animals (Pattij, 2002). The fact that HBK-17 normalized heart rhythm in adrenaline-induced arrhythmia might complement its anxiolytic-like effect.

Interestingly, in both behavioral tests, HBK-17 showed an inverted U-shaped dose-effect (only one dose was active). A similar dose-effect function was demonstrated for a reference compound, a selective 5-HT_7_ receptor antagonist, SB 269970 and a 5-HT_1A_ receptor agonist, 8-OH-DPAT in the four plate test. In the elevated plus maze test the only active dose of 8-OH-DPAT was the highest dose tested (i.e., 2 mg/kg). Conversely, a well-known anxiolytic, diazepam, showed a dose dependent-effect in both tests (Pytka et al., 2015a). Although scientists very often report such a non-linear relationship in pharmacological studies (Baldi and Bucherelli, 2005), an inverted U-shaped dose-effect it is very poorly understood. Some scientists suggest that this effect might have multifactorial nature, which is difficult to explain. In the case of HBK-17, there might be an explanation of this phenomenon. The anxiolytic-like effect of HBK-17 at higher doses might disappear due to the sedative properties of the compound, which we reported in our previous studies (ED_50_ = 15.0 mg/kg, observation time: 30 min) (Waszkielewicz et al., 2015). It is also possible that as HBK-17, 8-OH-DPAT, and SB 269970 showed an inverted U-shaped dose-effect, this might be a common feature of serotonin agents.

Toward a better understanding of the pharmacological properties of HBK-17, we evaluated the compound’s affinity for GABA_A_, adrenergic α_2_ and dopamine D_2_ receptors. The studied compound showed a moderate affinity for D_2_ receptors, whereas no affinity for GABA_A_ and α_2_ receptors. Our previous studies demonstrated that HBK-17 showed high affinity for α_1_ (Pytka et al., 2016a) and 5-HT_7_ (Waszkielewicz et al., 2015), moderate for 5-HT_2A_ (Kubacka et al., 2016), and very low for 5-HT_6_ and 5-HT_3_ receptors (Waszkielewicz et al., 2015; Kubacka et al., 2016). Bearing that in mind, we evaluated the compound’s intrinsic activity at 5-HT_2A_, 5-HT_7_, and D_2_ receptors. Our functional studies indicated that HBK-17 was a moderate D_2_ and very weak 5-HT_7_ receptors antagonist.

In our previous experiments, HBK-17 showed antagonistic properties at 5-HT_1A_ receptor (Ca^2+^ mobilization assay), in this study we demonstrated that the compound showed functional selectivity at the 5-HT_1A_ receptor. It preferentially activated ß-arrestin recruitment vs. p-ERK1/2, cAMP production inhibition or Ca^2+^ mobilization. Interestingly, HBK-17 showed partial agonistic activity in the p-ERK1/2 assay. Studies demonstrated decreased ERK expression and phosphorylation in post-mortem brains of depressed patients (suicide victims) (Dwivedi et al., 2001). Similar changes were observed in animal models of depression (Tiraboschi et al., 2004). Therefore, the fact that HBK-17 increased p-ERK1/2 might at least in part underlie its previously described antidepressant-like properties (Waszkielewicz et al., 2015; Kubacka et al., 2016). At this point, it is also worth mentioning that ß-arrestins desensitize not only G-protein-dependent signal pathways but also promote novel pathways of signal transduction, e.g., ERK, JNK, p38, or Akt (for review see DeWire et al., 2007). Thus, it might be possible that the observed increase in p-ERK1/2 might also be a result of the activation of ß-arrestin recruitment. Nevertheless, we need to perform more experiments to explain this issue.

To find the possible mechanism of anxiolytic-like activity of HBK-17, we performed another set of experiments, i.e., pretreated mice with pCPA (tryptophan hydroxylase inhibitor), WAY-100635 (non-selective 5-HT_1A_ receptor antagonist), or AMPT (tyrosine hydroxylase inhibitor). Imaizumi et al. (1996) demonstrated that a 3-day treatment with pCPA (200 mg/kg) significantly reduced serotonin levels in the cortex (30%), diencephalon (35%), midbrain (24%), and pons with medulla (34%). The depletion of serotonin levels by pCPA abolished the anxiolytic-like activity of HBK-17 in the four plate test. We observed a similar effect after pretreatment with WAY-100635, which implies that the anxiolytic-like activity of HBK-17 depended on the activation of the serotonergic system, and particularly 5-HT_1A_ receptors. In contrast, AMPT did not affect the compound’s activity in this test. This suggests that noradrenergic and dopaminergic systems are not involved in HBK-17 anxiolytic-like activity. We speculate that the anxiolytic-like activity of HBK-17 might have been a result of the postsynaptic 5-HT_1A_ receptors internalization induced by the recruitment of ß-arrestins and possibly the stimulation of presynaptic receptors. Nonetheless, our hypothesis requires confirmation.

Pharmacokinetic parameters of the compound influence its pharmacological activity. Thus, we evaluated the pharmacokinetic profile of HBK-17. We demonstrated that HBK-17 was rapidly absorbed with the peak concentration occurring after 5 min and had a rather long half-life of about 105 and 150 min when administered *i.v.* or *i.p.*, respectively. The volume of distribution (9.7 L/kg) suggests that the compound was moving throughout the body water. We should emphasize the relatively high bioavailability (44.8%) of the studied compound. HBK-17 could penetrate the brain tissue with the brain/plasma ratio of 1.13 and 0.96 after *i.v.* or *i.p.* administration, respectively. The results show that we could administer the compound by both *i.v.* and *i.p.* routes. Therefore, we conclude that HBK-17 has a desirable pharmacokinetic profile for pharmacological studies. Moreover, to our knowledge, this is the first report to quantify HBK-17 in biomatrices and assess its pharmacokinetics in mice.

## Conclusion

We demonstrated that HBK-17 possessed anxiolytic-like activity in mice, which depended on the 5-HT_1A_ receptor activation. The compound after binding to the 5-HT_1A_ receptor preferentially activated β-arrestin recruitment over Ca^2+^ mobilization, phosphorylation of ERK1/2 or cAMP formation inhibition. We also demonstrated that following a single *i.v.* or *i.p.* administration, HBK-17 showed relatively high bioavailability, rapid absorption to the blood but rather low distribution to brain. The interesting pharmacological profile of HBK-17 encourages further experiments to understand its mechanism of action fully.

## Author Contributions

KPy, JS, BF, and MK conceived and designed the experiments. KPy, MG-L, KN, AS, MS, KPa, EŻ, and KS performed the experiments. KPy, MG-L, MW, AS, AO, AG, and JŚ analyzed the data. MK, KPy, AW, and HM contributed to reagents, materials, and analysis tools. KPy, MG-L, KS, EŻ, MW, AS, and AW wrote the paper.

## Conflict of Interest Statement

The authors declare that the research was conducted in the absence of any commercial or financial relationships that could be construed as a potential conflict of interest.
